# Effect of Bleaching on Color Change and Surface Topography of Composite Restorations

**DOI:** 10.1155/2010/695748

**Published:** 2010-12-22

**Authors:** Gunjan Pruthi, Veena Jain, H. C. Kandpal, Vijay Prakash Mathur, Naseem Shah

**Affiliations:** ^1^Department of Prosthodontics, Center for Dental Education and Research, All India Institute of Medical Sciences, New Delhi, India; ^2^Optical Radiation Standards, National Physical Laboratory, New Delhi, India; ^3^Department of Pedodontics, CDER, All India Institute of Medical Sciences, New Delhi, India; ^4^Department of Conservative Dentistry and Endodontics, CDER, All India Institute of Medical Sciences, New Delhi, India

## Abstract

This study was conducted to determine the effect of 15% carbamide peroxide bleaching agent on color change and surface topography of different composite veneering materials (Filtek Z350 (3M ESPE), Esthet X (Dentsply India), and Admira (Voco, Germany). *Methods*. 30 samples were fabricated for evaluation of color change using CIELAB color system and Gonioreflectometer (GK 311/M, ZEISS). 45 disc-shaped specimens were made for evaluation of surface topography after bleaching (Nupro White Gold; Dentsply) using SEM. *Statistical analysis*. One way ANOVA and Multiple comparison tests were used to analyze the data. Statistical significance was declared if the *P* value was .05 or less. *Results and conclusion*. All the specimens showed significant discoloration (Δ*E* > 3.3) after their immersion in solutions representing food and beverages. The total color change after bleaching as compared to baseline color was significant in Filtek Z350 (*P* = .000) and Esthet X (*P* = .002), while it was insignificant for Admira (*P* = .18). Esthet X showed maximum surface roughness followed by Admira and Filtek Z350. Bleaching was effective in reducing the discoloration to a clinically acceptable value in all the three groups (Δ*E* < 3.3).

## 1. Introduction

Composite resin restorative materials undergo a series of physical changes as a result of the polymerization reaction and the subsequent interaction with the wet oral environment [1]. This process may cause softening of the resin matrix and reduction of stain resistance [2, 3].

Discoloration of tooth-colored resin-based materials may be caused by intrinsic and extrinsic factors. Intrinsic factors involve the discoloration of the resin material itself, such as alteration of the resin matrix and the interface of matrix and fillers [4]. Extrinsic factors such as adsorption or absorption of stains may also cause discoloration [5, 6]. Various authors have reported the staining of resin-based materials by coffee, tea, and other beverages [5–13], and color stability after aging in different solutions [4, 14–16]. Scotti et al. found out that the type of material also had significant role on the stain resistance [16]. Stober et al. reported that red wine and coffee caused severe discoloration, with total color differences of Δ*E* > 10 in all the tested composites [17]. Um and Ruyter stated that discoloration by coffee was due to absorption of colorants by the tested materials [18].

As discoloration of resin-based composites is a common problem, studies also investigated the effect of bleaching agents on tooth-colored materials [19–28]. Cooley and Burger evaluated composite resins for change in surface hardness, roughness, and lightness after exposure to 10% carbamide peroxide gels and found that these three parameters increased significantly after exposure [19]. Fay et al. observed that 10% carbamide peroxide used in the study removed stains from composite resin and hybrid ionomer but was not effective in removing them from the compomer [21].

New bleaching agents continue to appear in the market that can be used effectively in bleaching human teeth. The present study was conducted to evaluate the effectiveness of 15% carbamide peroxide bleaching agent on the color change and surface characteristics of three different composite veneering materials with different compositions and particle size: Admira (ormocer), Filtek Z350 (nanocomposite), and Esthet X (micromatrix composite) after experimentally inducing discoloration with tea, turmeric solution (a common ingredient in Indian cooking) and a carbonated soft drink (Coke).

## 2. Method and Materials

### 2.1. Test Materials

Three contemporary direct composites Filtek Z 350 (a nanoparticle composite), Esthet X (a microhybrid composite), and Admira (an Ormocer-based composite), were tested (Table 1). The effect of bleaching on color change and surface characteristics was evaluated after immersion in Coke (as available in market) and solutions of Tea and Turmeric. Fifteen percent carbamide peroxide gel (Nupro white gold, Dentsply); an out-office bleaching agent was used to evaluate the effectiveness of bleaching on the color of the composites.

**Specimen Preparation**

**Figure 1:**
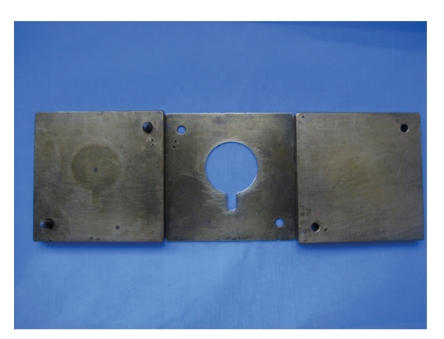
Three piece brass mould.

- For color evaluation, ten discs of each composite material were prepared with standard dimensions of 30 mm diameter and 1 mm thickness (A 2 shade), using three-piece brass metal mold with specified dimensions (Figure 1). These dimensions (30 × 1 mm) were taken to match the dimensions of the aperture of Gonioreflectometer, where disc was placed for color measurement.
- For evaluation of surface topography, a total of 45 discs were prepared in the same manner with standard dimensions of 1.5 × 1.5 mm and A2 shade and were equally divided into three groups. Ten discs of each material were kept for experiment and five discs of each material were kept as control.

#### 2.1.1. Composite Disc Fabrication over the Brass Mold

A cellophane sheet was placed at the base of mold and then the material was placed in the mold. Another cellophane sheet was placed on top of the material to achieve a smooth surface finish. Top part of the mold was placed over it and pressed uniformly with hand till all the three parts precisely approximated to each other. All the three parts of the mold were separated. Sample discs were light cured using LED light-curing unit (Blue phase C 5, Ivoclar Vivadent, light intensity—500 mW/cm^2^) from both the sides at 1 mm distance in four overlapping quadrants to cover the whole surface area for specified duration following manufacturers' instructions (Filtek Z 350—20 seconds, Admira—60 seconds, Esthet—X-20 seconds). The specimens were placed in distilled water at 37°C for 24 hours to ensure complete polymerization. Discs were removed from the mold. Extra flash material at the boundaries of the disc was removed with the help of a superfine airotor bur and the boundaries were finished and polished with Enhance finishing points and polishing cups, using Prisma Gloss composite polishing paste (Enhance System, Dentsply Caulk, U.S.A).

#### 2.1.2. Preparation of Staining Solution

Tea solution was prepared by adding one premeasured tea bag (Taj Mahal tea) into 150 mL of boiling distilled water and simmering the solution for five minutes. Turmeric solution was prepared by adding 1.5 grams of turmeric powder to 150 mL of boiling distilled water. The solutions were cooled to room temperature and used as such. Coke (Coca cola Ltd.) was used as available in market and its freshness was maintained by keeping it in airtight bottle.

#### 2.1.3. Method of Staining

The ten sample discs of one material were kept in test solutions one by one for a three hour period per solution, for 40 consecutive days. Turmeric and tea solutions were freshly prepared and changed daily whereas new bottle of soft drink (coke) was used daily. Sample discs were gently rinsed with distilled water during change over from one solution to another and air dried. Same cycle was repeated for the other two groups. Color changes were assessed after 40 days using Gonioreflectometer.

#### 2.1.4. Color Measurement

**Gonioreflectometer**

(GK 311/M, ZEISS) was used for color measurements. It is a device for measuring a bidirectional reflectance distribution function (BRDF). The “gonio” part of the word refers to the multidimensionality of the device. The device consists of a light source illuminating the material to be measured and a sensor that captures light reflected from that material.

Each individual disc was placed one by one on the aperture of Gonioreflectometer and was covered before color measurement. Color measurements were taken at three occasions, that is, baseline, after 40 days and after bleaching for a period of 14 days. The CIELAB color space (Commission Internationale de I'Eclairage L*, a*, b*) system was used for the color measurements. Three color values given denote L* (lightness), a* (red-green axis), b* (blue-yellow axis). The lightness (L*) ranges between 0 = black to 100 = white. Positive a* values represent red hues and negative a* values represent green hues. Positive b* values represent yellow hues and negative b* values represent blue hues. Its greatest advantage as a tool for representing and characterizing color is its greater uniformity; colors values on the three axes are distributed closely and linearly with respect to the human perception of color.

Color changes were calculated using the formula

(1) $$\begin{array}{cc} \Delta E & ={\left[\Delta L^{2}+\Delta a^{2}+\Delta b^{2}\right]}^{1/2}. \end{array}$$

A perceptible discoloration, that is Δ*E* > 1.0, was taken as acceptable up to the value of Δ*E* = 3.3 in subjective visual evaluations under optimal lighting conditions [10, 21].

During the study, composite resin samples were also assessed subjectively by visual examination to evaluate whether a clinically observable color change occurred.

#### 2.1.5. Method of Bleaching

The test specimens were subjected to bleaching process by applying 15% carbamide peroxide bleaching solution for 8 hours per day for 14 consecutive days at room temperature to simulate the out-office bleaching conditions. Bleaching agent was applied with the help of a cotton applicator on the same surface of the sample every time and the samples were kept in dark containers. After bleaching, the specimens were rinsed under running water for 1 minute to remove the bleaching agent, blotted dry, and stored in distilled water at room temperature till the next application. The specimens were again subjected to color testing.

#### 2.1.6. Evaluation of Surface Topography

Surface topography was evaluated under Scanning electron microscope (SEM). Five samples of each group served as control as they were not subjected to bleaching. Gold sputtering of all the samples was done before mounting on the stub of SEM and surface topography was recorded at 250x and 1000x magnification. The subjective assessment was used to compare the surface topography between control and experimental groups as shown in photomicrographs.

### 2.2. Statistical Analysis

A master chart was prepared in MS excel sheet and analyzed using Statistical Package for Social Sciences (SPSS 11.5). One-way ANOVA was used to analyze the significance of results within groups. Multiple comparison tests with Bonferroni correction was used for comparison of values amongst different groups and the study parameters were considered significant at 95% confidence level.

## 3. Results

After staining, all the groups had clinically significant discoloration (Δ*E* > 3.3).  Table 2 shows that after bleaching, the color of all the three materials returned to the baseline and was below clinically perceptible limit (Δ*E* < 3.3). 

The total color change after bleaching as compared to baseline color was significant in Filtek Z350 (*P* = .000) and Esthet X (*P* = .002), while it was not significant in Admira (*P* = .18) (Table 2). Multiple comparison test for intergroup comparison of total color change at different time intervals showed that this difference was not significant for all the groups except the difference of color after staining between Esthet X and Admira, which was found to be statistically significant (*P* = .013).

### 3.1. Effect of Bleaching on Surface Topography of Composites

**Figure 2:**
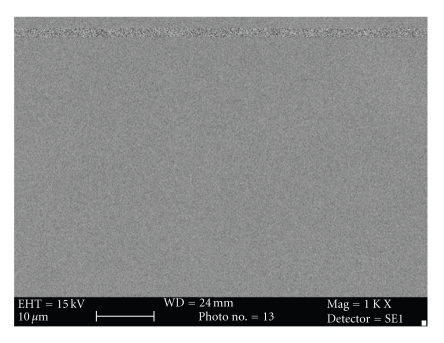
Photomicrograph of Filtek Z350 before bleaching at 1000x.

**Figure 3:**
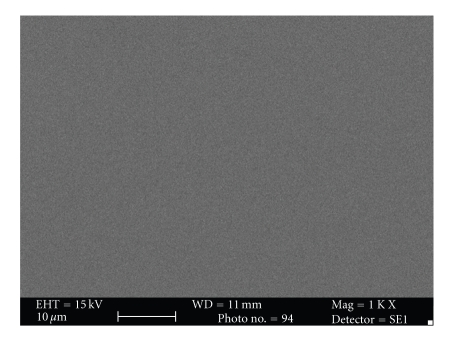
Photomicrograph of Filtek Z350 after bleaching at 1000x.

**Figure 4:**
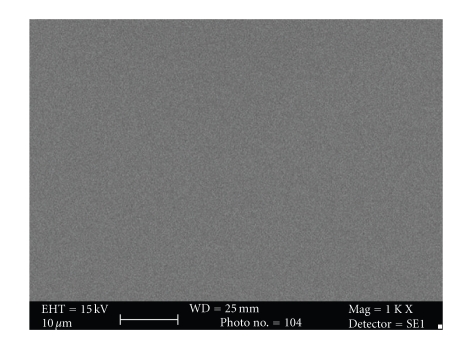
Photomicrograph of Esthet X before bleaching at 1000x.

**Figure 5:**
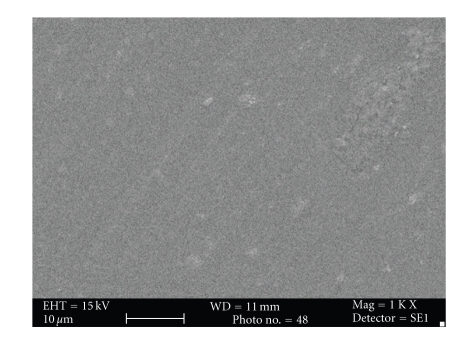
Photomicrograph of Esthet X after bleaching at 1000x.

**Figure 6:**
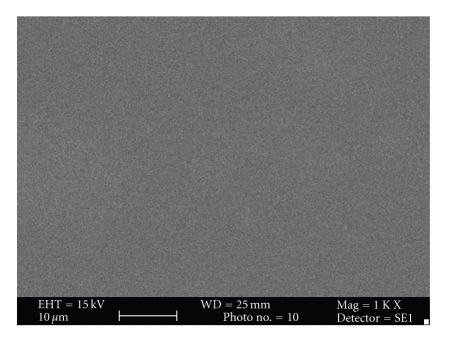
Photomicrograph of Admira before bleaching at 1000x.

**Figure 7:**
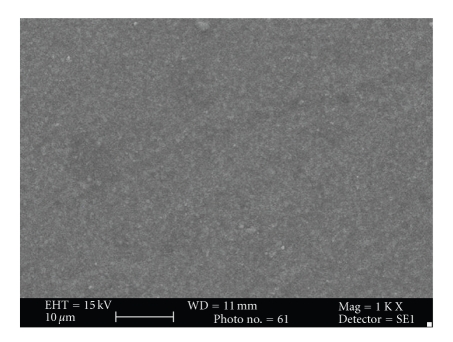
Photomicrograph of Admira after bleaching at 1000x.

 Subjective evaluation of surface topography of control and experimental group under Scanning electron microscope suggested increase in surface roughness of the samples in the experimental groups after the bleaching treatment. Surface roughness was maximum with Esthet X group (Figures 4 and 5) followed by Admira (Figures 6 and 7) and the least with Filtek Z350 (Figures 2 and 3).

## 4. Discussion

Dental restorative materials are exposed to saliva, stains, food components, and beverages in the oral environment [29]. Routine food habits can affect the esthetic quality of composite restorations. To ensure excellent aesthetics, it is necessary for tooth-colored materials to maintain intrinsic color stability and a resistance to surface staining. However, over time, composite restorations do acquire external stains and internal discoloration. This could be attributed to either material being biphasic; that is, composed of matrix and filler particles which provides scope of inclusion of external stains in its structure or various food items such as tea, coffee, tobacco, and turmeric (a common ingredient in Indian cooking), being saturated with colorants. 

Discolored composite restorations are unsightly necessitating replacement of the entire restoration, which is very expensive. The other cost-effective alternatives are either finishing and polishing or bleaching of the restoration. The interaction between the bleaching agent and restorative material is of clinical significance and therefore needs to be evaluated [25].

Spectrophotometers and colorimeters have been used to measure discoloration since they eliminate the subjective interpretation of visual color comparison [30–32]. The CIELAB system for measuring chromaticity was chosen to record color differences because it is well suited for determination of small color differences [33]. Various studies have reported different thresholds of color difference values above which the color change is perceptible to the human eye [32, 34–37]. ΔE* values >1 is considered to be visible to the naked eye, and ΔE* ≥ 3.3 is considered as clinically unacceptable [38].

### 4.1. Effect of Bleaching on Color Change

In the present study, a significant change in color was found in all the test materials after their exposure to discoloring solutions. Esthet X was affected the most (Δ*E* = 6.71) and Admira (Δ*E* = 4.42) the least after immersion in discoloring solutions (Table 2). Observations of previous studies reveal that the effect of staining solutions on color change of composite resins is material dependent and has been primarily attributed to basic composite formulation, type of filler particles and particle size [5, 39]. 

The type of resin matrix has been shown to play an important role in stain susceptibility of composites; for example, urethane dimethacrylate (UDMA) seems to be more stain resistant than bis-GMA [29]. Pearson documented that under normal curing conditions, a urethane dimethacrylate material showed lower water sorption than bis-GMA. Use of different proportions of diluents like TEGMA to modify the handling properties of the materials in bis-GMA resin can also affect its esthetic properties [40]. It is apparent that water sorption and solubility of resin composites is dependent on the type of resin matrix used [29]. The structures of the resin matrix of all the resin composites used in this study were different, Admira is an ormocer, Esthet X contains bis-GMA resin, and Filtek Z350 is a nanocomposite-containing urathane (Table 1). Ormocer with its rigid organic matrix containing 3-dimensionally linked inorganic-organic copolymers (ormocers) and additive aliphatic and aromatic dimethacrylates has high wear resistance as compared to microfilled or microhybrid composite and can resist discoloration. UDMA containing Filtek Z350 was found to be more resistant to the effect of discoloring solutions than Bis-GMA containing Esthet X. 

Staining susceptibility of resin composites is also attributed to the degree of water sorption and hydrophilicity of the matrix resin [41]. Excessive water sorption can increase the staining susceptibility of composite restorations [40]. If the resin composite can absorb water, then it can also absorb other fluids, resulting in its discoloration [41]. Water sorption decreases the life of resin composite by expanding and plasticizing the resin component, hydrolyzing the silane, and causing microcrack formation. These microcracks at the interface between the filler and the matrix allow stain penetration and discoloration. It has been shown that hydrophilic materials have a higher degree of water sorption and relatively higher discoloration value with staining solutions than hydrophobic materials [29].

Bis-GMA- and TEGDMA-based composites (Esthet X) are more hydrophilic than UDMA-based composites (Filtek Z350) or ormocers (Admira) because of the presence of hydroxyl group in their chemical structure. Consequently, dental materials that contain such monomers may be more susceptible to water sorption and discoloration over time.

Total change in color of all the test samples after their immersion in staining solutions was more than clinically acceptable limit (Δ*E* > 3.3). But bleaching was effective in reducing the discoloration in all the three groups to within the clinically acceptable value (Δ*E* < 3.3). The color change of composite resins after bleaching was probably due to superficial cleansing of the specimens. The results of previous studies as well as this study indicate that the color change induced by the bleaching agent might be dependent upon the monomer structure, and volume of the resin matrix as well as the filler systems of composite materials [42].

### 4.2. Effect of Bleaching on Surface Topography

Subjective assessment of SEM images after bleaching revealed increase in surface roughness of all the materials. Esthet X was affected the most (Figures 4 and 5) and Filtek Z350 (Figures 2 and 3) the least. Previous studies have shown that carbamide peroxide bleaching gels may lead to slight roughness of resin-based composites although it may have no clinical significance [19, 20]. 

It has been found that bleaching agents impair the surface integrity, affecting the penetration depth of the bleaching agent [42]. In the present study, SEM images revealed that all the composites tested underwent surface alterations of their superficial surface after bleaching. Interestingly, some studies have reported an increase [19], decrease [20], or unchanged [28] composite surface after applying carbamide peroxide gels for varying time periods.

In the present study, Esthet X showed maximum surface roughness followed by Admira (Figures 6 and 7) and Filtek Z 350 in the decreasing order. The results of previous studies had suggested that the surface of the micro hybrid resin composite reveals more irregular features and seems to be rougher in comparison to ormocer and microfilled composites. The large particle size in microhybrid composites can enhance microporosity in the structure [42]. This can explain the reason for least change in surface topography of Filtek Z350 after bleaching as compared to the other two composites as it has smallest filler particle size (20-nm). The filler size of Esthet X is 0.6–0.8 *μ*m, while that of Admira is 0.04–1.2 *μ*m (Table 2). The largest filler size of Esthet X amongst all the groups explains its maximum rough surface after bleaching. Although many other factors like polishability of the material, technique used for finishing and polishing, materials used for polishing, type and concentration of the bleaching agent used can affect the surface topography of a material, these factors were kept uniform and constant for all the test materials within the limits of experiment. Surface of these discs was not finished or polished to attain uniformity and only one type of bleaching agent with a specific composition was used. The most important factor considered responsible was the inherent differences in the composition of the materials used.

## 5. Conclusions

It is concluded that there was a significant color change in all the test groups after their immersion in various staining solutions and the discoloration was more than clinically acceptable value (Δ*E* > 3.3). After bleaching, ΔE returned to clinically acceptable limits (Δ*E* < 3.3). The difference in behavior amongst various groups was basically due to the difference in their inherent compositions. The surface roughness in all the test materials was minimal when seen under SEM after subjecting them to bleaching treatment. 

As true for all in vitro studies, the constraint of reproducing accurate clinical conditions also applies to the present study.

## Figures and Tables

**Table 1:** Physical characteristics of experimental materials.

Material	Filtek Z350	Esthet X	Admira
Manufacturer	3M ESPE	Dentsply	Voco, Germany

Type	Nanocomposite	Microhybrid	Ormocer based

Organic matrix	Combination of a non-agglomerated/non-aggregated, 20 nm nanosilica filler, and loosely bound agglomerated zirconia/silica nanocluster, consisting of agglomerates of primary zirconia/silica particles	Resin matrix (a urethane-modified Bis-GMA) is based on the matrix used in TPH Spectrum	3-dimensionally linked inorganic-organic copolymers (ormocers) and additive aliphatic and aromatic dimethacrylates

Filler type	Bis-GMA, UDMA, TEGDMA, and Bis-EMA resins	Barium alumino fluoroborosilicate glass (BAFG) and nanosized silicon dioxide particles	Ba-Al-B-silicate glass

Average particle size	0.6–1.4 microns	0.02 to 2.5 microns (with an average of from 0.6 to 0.8 microns)	0.04–1.2 *μ*m

Filler volume %	78.5% by wt/59.5% by vol	60% by volume	56% by volume/78% by wt

**Table 2:** Total discoloration (Δ*E*) for all test samples at three different time intervals using one way ANOVA and multiple comparison test of total discoloration (Δ*E*) between different test materials using Bon Ferroni correction.

Group	Baseline- staining Δ*E*_1_	Staining-bleaching Δ*E*_2_	Bleaching-baseline Δ*E*_3_	Intragroup significance value (Δ*E*_3_ versus Δ*E*_1_)
Filtek Z350	6.3 (1.71)*	5.76 (1.19)	1.97 (1.39)*	*P = .000
Esthet X	6.71 (1.98)**	6.58 (1.77)	1.79 (1.35)**	**P = .002
Admira	4.42 (1.10)^NS^	6.79 (2.09)	3.02 (1.6)^NS^	^NS^ *P* = .18

Esthet X versus Admira—*P* = .013. Significant = *P* < .001, NS = *P* > .05

The calculations of Δ*E* were as follows:

Δ*E*_1_ = [(Δ*L*2−Δ*L*1)^2^+(Δ*a*2−Δ*a*1)^2^+(Δ*b*2−Δ*b*1)^2^]^1/2^

Δ*E*_2_ = [(Δ*L*3−Δ*L*2)^2^+(Δ*a*3−Δ*a*2)^2^+(Δ*b*3−Δ*b*2)^2^]^1/2^

Δ*E*_3_ = [(Δ*L*3−Δ*L*1)^2^+(Δ*a*3−Δ*a*1)^2^+(Δ*b*3−Δ*b*1)^2^]^1/2^
